# ‘ *It is a question of determination’*: a case study of monitoring and evaluation of integrated family planning services in urban areas of Togo

**DOI:** 10.12688/gatesopenres.12944.1

**Published:** 2019-05-01

**Authors:** Helen Baker, Roger Rochat, Kenneth Hepburn, Monique Hennink, Macoumba Thiam, Cyrille Guede, Andre Koalaga, Eloi Amegan, Koffi Fombo, Bolatito Ogunbiyi, Lynn Sibley

**Affiliations:** 1 Nell Hodgson Woodruff School of Nursing, Emory University, Atlanta, Georgia, 30322, USA; 2 Rollins School of Public Health, Emory University, Atlanta, Georgia, 30322, USA; 3 AgirPF, EngenderHealth, Lomé, Togo; 4 EngenderHealth, Washington D.C., 20004, USA

**Keywords:** Postabortion family planning, Postpartum family planning, Monitoring and evaluation, Togo, West Africa

## Abstract

**Background:** Integrating family planning into postabortion and postpartum services can increase contraceptive use and decrease maternal and child death; however, little information exists on the monitoring and evaluation of such programs. This article draws on research completed by the EngenderHealth’s *AgirPF* project in three urban areas of Togo on the extent to which monitoring and evaluation systems of health services, which operated within the *AgirPF* project area in Togo, captured integrated family planning services.

**Methods:** This mixed methods case study used 25 health facility assessments with health service record review in hospitals, large community clinics, a dispensary, and private clinics and 41 key informant interviews with health faculty, individuals working at reproductive health organizations, individuals involved in reproductive health policy and politics, health care workers, and health facility directors.

**Results:** The study found the reporting system for health care was labor intensive and involved multiple steps for health care workers. The system lacked a standardized method to record family planning services as part of other health care at the patient level, yet the Ministry of Health required integrated family planning services to be reported on district and partner organization reporting forms. Key informants suggested improving the system by using computer-based monitoring, streamlining the reporting process to include all necessary information at the patient level, and standardizing what information is needed for the Ministry of Health and partner organizations.

**Conclusion:** Future research should focus on assessing the best methods for recording integrated health services and task shifting of reporting. Recommendations for future policy and programming include consolidating data for reproductive health indicators, ensuring type of information needed is captured at all levels, and reducing provider workload for reporting.

## Introduction

Interest in providing family planning integrated into other health services has increased in the last five years ^1– 3^. While it is well established that integrating services such as family planning into postabortion and postpartum care can increase contraceptive use and decrease maternal and child deaths ^2, 4^, little information exists on the monitoring and evaluation of such programs at country or regional levels ^5^.

Two studies in Togo, the focus of this paper, on postabortion care (PAC) and record keeping at five participating health facility sites documented that in 2014 the monitoring systems for PAC were informal and non-standardized in four of the five facilities ^6^. With further training and support, a follow up study in 2016 in the same sites found standardized PAC registers which were generally filled out at the patient level, but the information was not transferred to the district, regional, or national levels ^7^.

In opposition to this lack of standardized monitoring, there is increasing interest in monitoring progress towards international benchmarks such as the Sustainable Development Goals, as well as indicators for health initiatives funded by international organizations. Some of the barriers to timely and accurate monitoring include a lack of funding and resource allocation to monitoring and evaluation ^8^, a disconnect between expectations of high quality monitoring systems and the level of detail required in reporting ^8– 10^, poor linkages between monitoring systems and points of data generation ^11^, a shortage of trained professionals working in monitoring and evaluation ^12– 15^, and the large quantities of required health indicators ^8^, which often results in duplication of data collection, and frequent underutilization of existing data collection tools ^16, 17^.

The best methods for reporting integrated family planning programs in practice are still being developed, especially in areas where health care is highly fragmented by type of service delivery (such as vaccination, maternity care, HIV care) ^18– 20^. The published literature lacks information about the processes and strategies for reporting integrated family planning services when implemented ^4^.

### Project description and study aims

*Agir pour la planification familial ( AgirPF)* was a 5-year USAID/West Africa project (2013–2018) implemented by EngenderHealth to build capacity in and increase access to family planning, with interest in the integration of family planning into PAC and postpartum services. *AgirPF* was implemented in the urban and peri-urban areas of five West African countries- Burkina Faso, Cote d’Ivoire, Mauritania, Niger, and Togo, in partnership with ministries of health, the private sector, and non-governmental organizations (NGOs).

In this paper, we describe the extent to which monitoring and evaluation systems of health services, which operated within the *AgirPF* project area in Togo, captured integrated family planning services. We then examine the influence of values, interests, and power dynamics between key stakeholders on quality of the monitoring and evaluation system and discuss the implications for policy, programming, and research. The paper is based on research that was conducted as part of a larger case study ^21^ completed by EngenderHealth’s *AgirPF* project in April-August 2016 with the goal of understanding the status of integrated family planning in urban areas of Togo.

## Methods

### Study design and setting

For this study we used mixed methods including health facility assessments, health service record review, and key-informant interviews. The study was situated in the three largest urban areas in Togo, Lomé (pop. 956,000), Sokodé (pop. 114,800), and Kara (pop. 110,900) ^22^. Between February and June 2016, and prior to data collection for this study, *AgirPF* initiated training around the integration of family planning with other health services. They trained health care workers at 35 facilities in PAC/postabortion care-family planning (PAC-FP), and health care workers at 7 health facilities in postpartum intrauterine device (PPIUD) insertion. 

### Procedures

***Health facility assessment.*** We obtained a diverse, purposive sample of 25 health facilities affiliated with *AgirPF* including university hospitals (n=2), regional hospitals (n=3), district hospitals (n=5), large community health facilities (n=9), a health dispensary (n=1), and private clinics (n=5).

For the assessment, we adapted the *Postabortion Care-Family Planning Service Availability and Readiness Assessment* ^23^ and ISSU *Enquête Finale au Niveau des Structures de Santé* (Final Survey at the Health Facility Level) *2015* ^24^.The adapted guide contained 194 both open and closed-ended questions. For this part of the health facility assessment, we focused on 19 questions related to monitoring and evaluation of reproductive, sexual, and child health services, and reporting of integrated family planning services.

We trained a data collection team of two nurses and four midwives and pilot tested the guide in July 2016. During the pilot testing and first 3 health facility assessments, the research team discovered that health workers were adapting the government approved and widely used family planning health registers to capture additional aspects of health service integration. This included recording information about what other services the woman received (PAC, postpartum care, immunizations, child health) in addition to whether the woman came on her own or with her husband/male partner. The health facilities also were creating their own unofficial registers to capture information about PAC and general gynecological care.

With this discovery, the health facility assessment team was asked to photograph de-identified filled out health service registers, including family planning, during the health facility assessments. These registers illustrate the different ways in which the registers had been adapted when the official register did not provide a way to capture information needed for reporting to the Ministry of Health and other agencies, including international NGOs.

The team conducted the assessments in person at the selected health facilities. They spoke with a staff member who had been designated by the director of the health facility to participate in the assessment. Since taking photos of the health facilities was not in the original study plan, the data collectors took photographs of different types of available health registers (e.g. not in use or inaccessible), and if the team member had use of a digital camera or smart phone with a camera the day of the health facility assessment. On average the health facility assessments took about three hours to complete by one data collector.

We entered all the closed ended data from the health facility assessment questionnaire into SPSS 24 ^25^ software for cleaning and analysis and compiled the responses from the open-ended questions in tables in MS Word ^26^. We used univariate analysis to generate descriptive statistics related to information about reporting on integrated family planning and the services offered at the facility related to integrated family planning. The principal investigator (first author of this article) developed a simple guide to assist with the review and analysis of the photographs by record type.

***In-depth interview.*** We purposively sampled 41 total respondents from diverse professional roles within the Ministry of Health, academic institutions, NGOs/international organizations, and health services. These included faculty at schools of medicine, nursing, and midwifery (n= 3); directors and health workers in health services (n=9 and n=14, respectively); individuals working at reproductive health NGOs/international organizations (n=9); and individuals who worked primarily in reproductive health policy and politics (these included individuals working in the Ministry of Health and NGO workers focused on reproductive health policy) (n=7). This diversity in the sample was intended to elicit a variety of perspectives on reproductive health care from individuals with varying degrees of contact with the *AgirPF* program activities and trainings related to integrated family planning services. Respondents were recruited by *AgirPF* through official collaborations with the Togolese Ministry of Health, other reproductive health NGOs, and health facilities. 

We developed and pretested five semi-structured interview guides appropriate to the type of respondent. Each of the guides included questions for respondents about multiple different aspects of family planning and integrated family planning in Togo. This paper uses data from the key-informant interviews related to questions about monitoring and evaluation of family planning and integrated family planning.

Five Togolese social scientists and the principal investigator conducted the face-to-face audio-recorded interviews in French. The interviews took place at a time and location chosen by the respondents and took from 30 to 150 minutes to complete. Interview recordings were transcribed verbatim in French by the Togolese social scientists using Express Scribe software version 5.000 ^27^ and then copied into MS Word 2013 ^26^.

We entered the interview transcripts into Nvivo 11 for analysis ^28^ and then developed initial codes as well as an initial codebook with the input of Togolese social scientists. The principal investigator then coded all transcripts using the codebook and applied thematic analysis, a rigorous, inductive set of procedures with the goal of identifying and examining themes from textual data in a way which is transparent ^29^. Matrices were created in Nvivo to better understand the intricacies of the responses by participant type and location for codes related to monitoring and evaluation of reproductive and sexual health and emerging methods of recording family planning integrated into postabortion and postpartum care. Transcripts were then re-read for further understanding of themes identified, and quotes were chosen to further illustrate selected themes.

### Ethical review and informed consent

The study was approved by the Institutional Review Board at Emory University (eIRB#88781), the EngenderHealth Knowledge Management, Monitoring and Evaluation, and Research unit, and the Togolese Ministry of Health *Comité de Bioéthique pour la Recherche en Santé (CBRS) (AVIS N ^o^015/2016/CBRS du 30 juin 2016)*. Data were collected only after written informed consent was taken from participants using standard disclosure procedures.

## Results

The results are presented beginning with the health facility assessment and health record review followed by the results from the key informant interviews.

### Health facility assessment

***Description of the facilities.*** There were 13 facilities in Lomé, four in Sokodé, and eight in Kara, a total of 25 sampled facilities. Eleven of the facilities provided PAC prior to the start of the *AgirPF* project in 2013. In 2016, 24 of the facilities had some of their health care workers trained by *AgirPF* in PAC and PAC-FP, 6 had health care workers trained in postpartum family planning (PPFP) and PPIUD insertion. All the health facilities were supported in some way by the *AgirPF* project. Table 1 shows the types of reproductive and child services available at each health facility with an associated official government issued register or with an unofficial register made by each health facility.

**Table 1.** Types of services provided, presence of reporting guidelines, and reporting tools for integrated services.

	University Hospital (n=2)	Regional Hospital (n=3)	District Hospital (n=5)	Large community clinic (n=9)	Dispensary (n=1)	Private (n=5)	Total (n=25)
Types of services with associated reporting register							
Family planning	2	3	5	9	1	5	25
Postabortion *	2	3	5	8	1	5	24
Prenatal	2	3	5	9	1	5	25
Labor/delivery	2	3	5	9	1	3	23
Postpartum	2	3	5	9	1	5	25
Child health	2	3	5	8	1	4	23
Vaccination	2	3	5	9	1	5	25
Presence of guidelines for reporting integrated services							
Yes	0	0	2	5	1	2	10
Observed or reported records on family planning integrated into							
Postabortion care *	2	3	4	7	1	4	21
Immediate postpartum	2	1	4	6	1	3	17
Postnatal care	0	1	5	8	1	2	17
Infant care	0	1	2	7	0	1	11
Vaccination	0	1	5	4	0	1	11

*Italic indicates an “unofficial” register

### System of reporting reproductive and child health services

**Figure 1.**
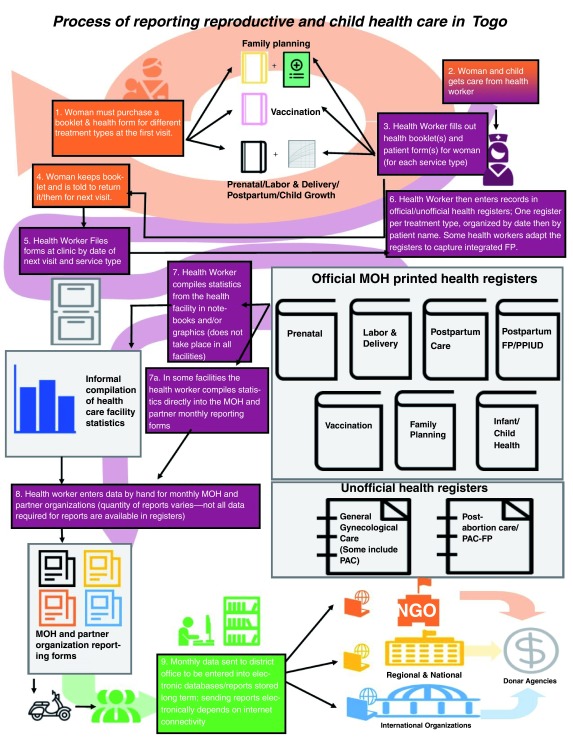
The process of Reporting Reproductive and Child Health Care in Togo. The expected data collection steps and tools used in the reporting process to monitor utilization of integrated family planning services in Togo.

***The expected system.*** The expected flow of information in the system is from the patient to the health facility to the district, regional, national, and international levels of the Ministry of Health ( Figure 1). At the patient level, the health care worker must enter and then re-enter information multiple times about a single patient. For information to flow from the patient to facility levels, the health worker must initially enter information into a patient booklet and, for family planning and vaccination, into a patient form for these services. Then the health worker must file the patient form(s) and enter the information in the patient health booklet and sometimes a patient health form into one or more official health register(s). The PPFP/PPIUD register was the only official register which included information about integrated family planning services. This register also had a corresponding book with carbon copy sheets for the monthly reports sent to the Ministry of Health and the partner organizations.

**Figure 2.**
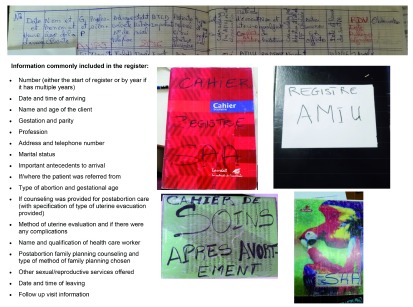
Examples of PAC Unofficial Registers with commonly included reported information. Common information collected by the Togolese Ministry of Health’s registers and images of the various registers utilized by family planning services reporting system.

***The actual system: individual level reporting.*** If an official register did not exist for a given service, sometimes NGO workers involved in integration projects would tell health workers to create an unofficial register for the service using a notebook and pen, inserting columns for recording information. Unofficial registers were used most often for general gynecological care (which sometimes included PAC/PAC-FP) as well as PAC/PAC-FP registers. There was little consistency across sites – they were idiosyncratic and site-specific in the unofficial records across the sampled facilities with respect to titles and the information captured. Figure 2 shows the different examples of PAC registers in use and a list of commonly included information in the PAC register.

**Figure 3.**
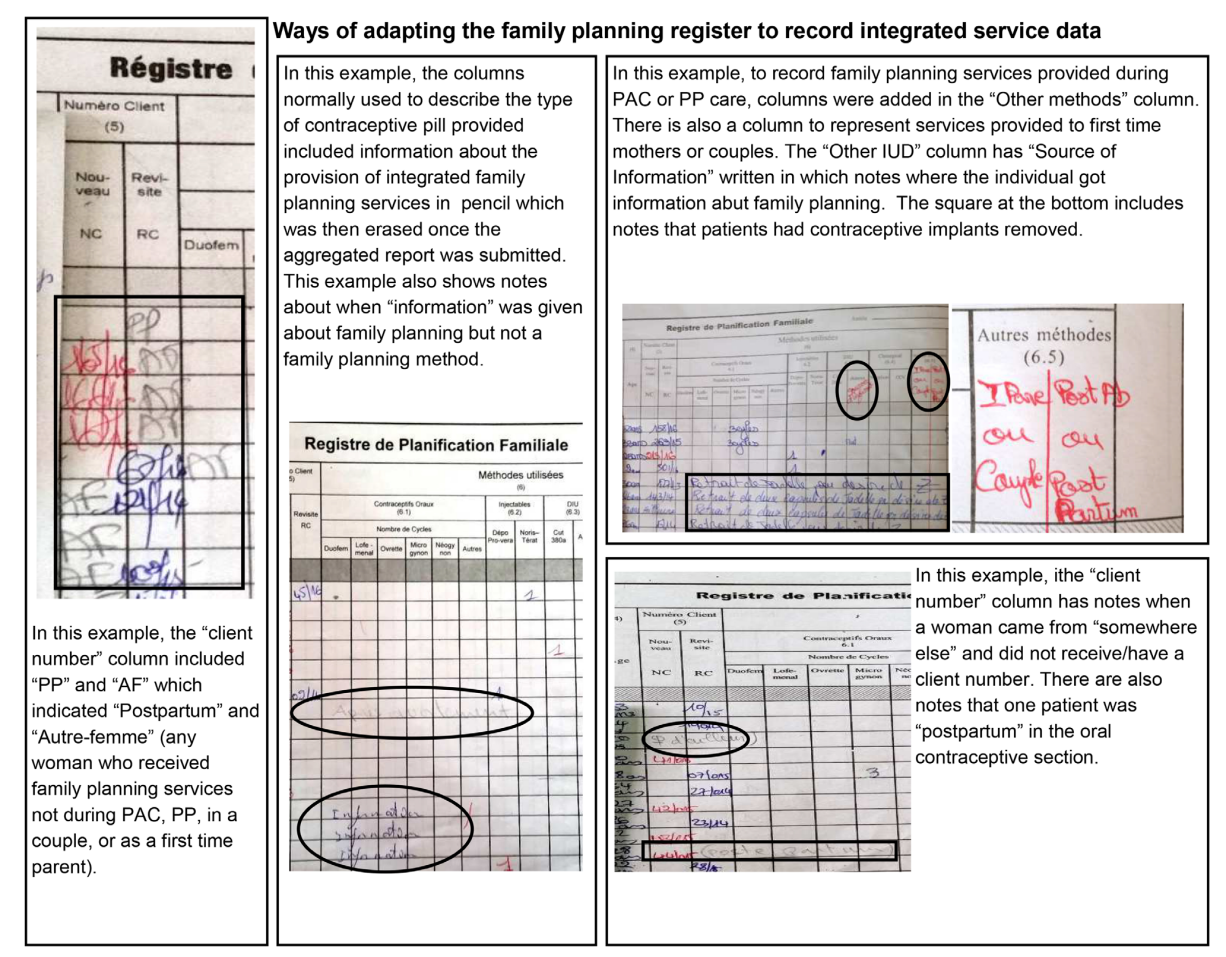
Ways of adapting the family planning register to record integrated services. How health workers adapted registers to capture necessary family planning services information necessary to report to the Togolese Ministry of Health and NGOs.

Family planning registers were adapted to capture when a client received family planning as part of other health services to enable the health workers to include these data on integrated health services into the monthly service provision reports ( Figure 3).

***The actual system: facility-level reporting.*** Within the past two years, the Ministry of Health revised its monthly service provision reporting form, *Maternal and Infant Health Report,* to include integrated family planning services as outlined in Table 2. However, the existing service registers were not changed accordingly. As a result, the registers did not always have the data needed to complete the new form at the facility level.

**Table 2.** Categories of information collected monthly in the revised “Report of Maternal and Infant Health” form.

• Women receiving family planning by method type • Women receiving prenatal care • Women with a referral to a high level of care due for pregnancy services • Deliveries by method of delivery and complications from delivery • Women receiving postnatal visits • Women who received a family planning method immediately after delivery • Women who received a PPIUD • Neonatal and maternal deaths • Women who received abortions by type (spontaneous or provoked) • Women who received PAC and PAC-FP • Women who received emergency obstetric care • Availability of emergency obstetric care • Availability of staff trained in emergency obstetric care

In addition to reporting at facility level for the Ministry of Health, the health care workers were also required to report monthly to the *AgirPF* project using *AgirPF’s* form. The respondents also prepared reports for other national and international organizations when asked.

**Figure 4.**
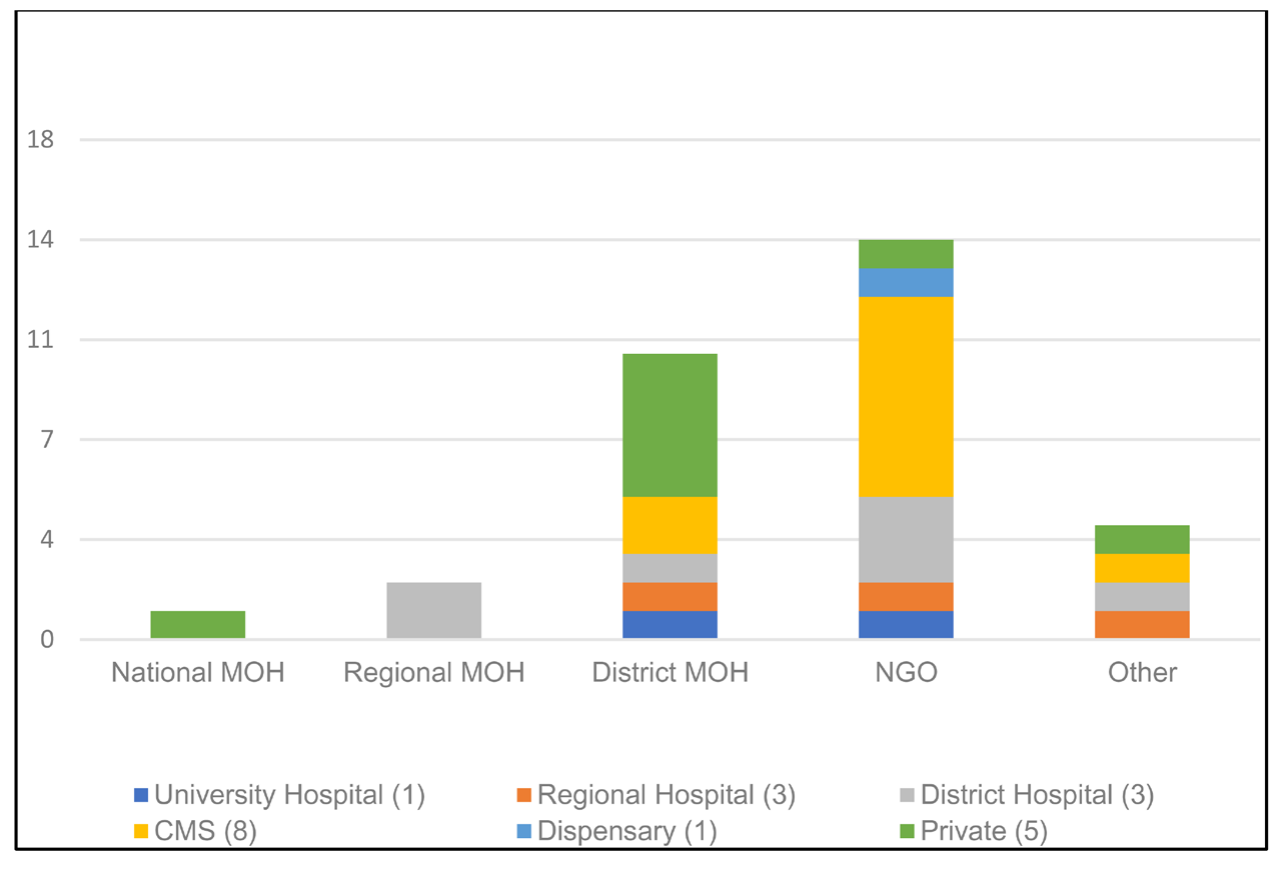
Where reports are sent each month by the sample of health facility. Where sampled health facilities send monthly monitoring reports and discrepancies between these facilities with respect to reporting up the chain.

The greatest discrepancy between the sampled facilities with respect to reporting up the chain ( Figure 1) was when and where the facility level reports were sent. All 21 of the health facilities which sent reports on integrated family planning sent them monthly. Figure 4 shows the recipients of reports sent by the health facilities. The most common responses included NGOs and the district level Ministry of Health; reports could be sent to more than one recipient. 

### Key informant interviews

Table 3 gives information about the 41 key informants involved in the study. Two of the health faculty were trainers for PAC, PAC-FP, PPFP, and PPIUD for the *AgirPF* project. One health facility director was trained in PAC/PAC-FP and 3 were trained in PPFP/PPIUD. One of the health care workers was a trainer for PAC and 8 had been trained in PAC. Four of the health care workers had been trained in PPFP/PPIUD insertion.

**Table 3.** Characteristics of key informants.

Characteristic	Category of informant				
	Health faculty (n=3)	Health facility directors (n=10)	Healthcare workers (n=14)	NGO workers (n=8)	Policy makers (n=7)
**Age (Mean, SD)**	49.3 (15.0)	45.1(10.8)	38.8(8.7)	53.2(6.2)	50.3(9.1)
**Sex (%)** Male Female	66 33	70 30	14 86	75 25	29 71
**Medical training (%)** Doctor Physician assistant Midwife Nurse Midwifery assistant No medical training	33 33 33 0 0 0	100 0 0 0 0 0	0 21 65 7 7 0	50 0 12 0 0 38	14 14 57 0 0 14
**Location of work (%)** Lomé Sokodé Kara Lomé & Kara	0 33 0 66	50 20 30 0	43 29 29 0	63 25 13 0	71 14 14 0
**Type of work place** Academic institution University Hospital Regional Hospital District Hospital Neighborhood Clinic Dispensary Private Clinic International Org International NGO National NGO National MOH Regional MOH	100 0 0 0 0 0 0 0 0 0 0 0	0 20 30 10 10 0 20 0 0 0 0 0	0 14 21 14 21 7 21 0 0 0 0 0	0 0 0 0 0 0 0 13 25 63 0 0	0 0 0 0 0 0 0 0 14 0 43 43
**Years working in RH** **(mean, SD)**	22.7 (12.7)	107.0 (11.0)	12.0 (7.4)	20.1 (11.3)	19.0 (8.5)

*One key informant was interviewed as both a faculty and health facility assessment.

***How family planning registers are being adapted to include integrated family planning data.*** The health workers interviewed conveyed a great interest in ensuring high quality reporting within the health care registers. What they noted as missing was appropriately formatted registers--both official and unofficial-- which captured all the information required in the facility-level monthly reports sent to the district level of the Ministry of Health and partner NGO projects. This most frequently occurred with the family planning register. One of the educators (02) who also worked as a clinician in a district hospital noted, “ *Some indicators that we must track are not noted in some registers, which is worrisome and causes extra work. The registers we have do not conform to the reporting tools. I suggest we review all the registers in use for family planning service providers and make sure they are consistent with the reporting tools. Then reporting would be easy.”*

***Availability of support for integrated family planning reporting.*** Individuals involved in reproductive health politics, NGOs, and health facilities directors had varying responses in relation to available support and resources to help implement and record family planning integrated with other health services. All but two respondents to this question felt that further support was necessary and that currently the availability of resources for integrated reporting was lacking, in part because the formats were not as useful as they could be, as described above. A major theme across respondents was how health care workers were already overwhelmed with the amount of required documentation that they were expected to produce. Respondents indicated that future reporting needed to be simplified or it would be necessary to have individuals specifically tasked with reporting. Training of health providers was a prominent request in improved reporting methods.

International NGOs provided health workers with the support for documentation of integrated services. During the PPFP/PPIUD training conducted by Jhpiego and EngenderHealth, health care workers being trained were given hard cover, bound registers approved by the Ministry of Health to record PPFP/PPIUD insertion to use at their respective health facilities as discussed above. One individual involved in reproductive health politics (05) said, “ *For PPIUD, Jhpiego provided a register for postpartum care! But postabortion care currently does not even have a proper collection tool. It is handmade and could be used at all levels but is not.”*

***Uses of family planning data that is reported.*** There was not consensus as to who used the collected family planning data from the informants. Informants named the Ministry of Health, the Division of Family Health/Division of Maternal and Infant Health/Family Planning, funding organizations, general government, their superior at the health facility, NGOs, United Nations agencies, and/or that it was used at all levels of the health system. Only 6 of 41 informants (three health care workers, three health facility directors) noted that they used the collected data from the health facilities themselves to inform their work and programming.

***Challenges and possible solutions associated with reporting integrated family planning services***

*1. Too many different registers*

Adding registers for integrated family planning was the most common way of recording these services. Two informants noted that there were numerous registers for all different types of health care areas and that there may be a point at which the number of registers was becoming excessive. One director (08) noted, “ *There are too many registers to fill out, but to get all the integrated services information one is tempted to increase the number of registers. On the other hand, we must rationalize to avoid having too much data to fill in. […] Integrated family planning must also be part of the overall data collection plan that is being worked on at the Ministry of Health.*”

Possible solutions suggested for this problem included bringing in support staff to help the health care workers to complete the reporting or, as noted above, revision and streamlining of relevant health reporting tools. One individual involved in reproductive health policy (01) said, “ *It could be helpful if there are specialized services for data collection as well.*”

*2. Lack of a standardized reporting system*

The need for standardized tools was noted by many of the informants. Without the proper tools to measure the integration of services it is impossible to know if the services are currently being integrated. An individual from a reproductive health NGO (05) said, “ *In terms of integration, data collection needs to have integrated tools, […] which must be at all levels of [patient care] delivery.”* Possible solutions for this were given by two informants (a health worker and a director) which included having the government officials learn more about reproductive health work needs and implementing electronic systems. One director wanted to make all the reporting in a computerized format, possibly using electronic tablets for providers to enter all data at the patient level.

*3. Difficulty in getting access to the registers*

More integration of registers took place in smaller health facilities compared to larger hospitals due to the geography of the hospitals and the separation of departments for PAC, labor and delivery, vaccination, and family planning. In the larger hospitals, the different gynecological services were offered in different areas by individuals trained specifically in that area, so the health care staff who oversee the family planning programs were not the ones who were running the PAC services. The hours of service availability also varied depending on the type of service. It was difficult to get the necessary access to these different areas within the larger health facilities to fill out the appropriate register. Often the person doing the PAC service had to go to the family planning service the following day to enter the information about the patient who had received family planning as part of PAC. This person was not necessarily identified specifically as having received family planning as part of PAC unless someone compared the family planning register with the PAC register and identified the woman by name.

In the smaller health facilities, one or two health care providers were responsible for all reproductive health services. This made it easier for them to record the integrated services since they were the ones providing all the services in one location. This way the registers were kept in one area and the health worker had the ability to choose how that register was used and how integrated services were noted.

According to informants, possible solutions to this included having the family planning register available in common locations which all health care workers have access to or change to electronic report (using computers or tablets) on services at the patient level in a platform such as DHIS2. The NGO workers wanted to have further exploration into how the problem of register location could be improved with greater knowledge of the work flow.

*4. Too much to record and too little time to do it*

Health care workers in Togo provide hundreds of services each month and are required to also chart these services, often in multiple charts and registers by hand. An individual involved in reproductive health politics (01) said, *“[Data collection] is done by service providers and they have a lot of work. The providers have to be providers, and I do not know, computer scientist, logistician, as well - they do everything at the same time.”* The informants mentioned the large number of tasks health care workers were responsible for throughout the interviews.

Possible solutions posed to this included having less information for the providers to fill out and adapt the reporting tools to streamline the process. One NGO worker (06) said, *“We need to have well-designed reporting tools that are not overloaded because […] when there are too many registers to fill in that can cause fatigue. We must […] adapt reporting tools and train personnel in the use of these tools.”*

***Why family planning reporting matters.*** The informants agreed that recording family planning played an important role in influencing future service needs. The areas which were prominent included the necessity to be able to make decisions and predict future service use; the ability to monitor improvements or declines in service, and if established goals were met; a way to justify staffing and expenses; and a way to see if there were current unmet needs or new program needs. An informant who worked at a reproductive health NGO (01) noted that while not always used effectively, “ *data allows us to know if we are moving towards the goals.”*

While there are many challenges to the reporting system, one facility director (07) made a thought-provoking statement when he said, in relation to improving reporting systems in Togo, “ *It seems like dreaming, but it is already done elsewhere, and it is a question of determination – we can do this if enough people think it is useful*.”

## Discussion

Reducing maternal and newborn morbidity and mortality is a priority of the Togolese government ^30^. Integration of family planning into other reproductive health services may help increase the modern contraceptive prevalence rate and decrease the unmet need for contraception which can contribute to reducing maternal and newborn mortality and morbidity. Quality data (e.g. timely, complete, precise and accurate) are key to being able to accurately measure the success of integrated services ^31^.

The study findings highlight vulnerabilities with respect to data quality in each link of the reporting chain at the individual patient and facility levels. These findings are unfortunately common. Numerous studies have highlighted the many challenges to reliable and timely information related to health service and health status of the population including problems with completeness, accuracy, and timeliness in low resource settings ^32, 33^, duplicate or parallel reporting systems and lack of capacity for data analysis ^34^ making planning, monitoring, and evaluation of these programs difficult ^35^. In the end, a health monitoring and evaluation system that is designed (unintentionally) to generate poor quality data provides a shaky foundation for health service decision-making, planning, and health policy.

### Global discussions around challenges and improvements to reproductive health monitoring and evaluation

Recording information about different types of care in individual registers is not unique to Togo and has been noted in other sub-Saharan African countries ^33, 36^. This type of recording makes it difficult to assign individual identifiers and increases staff workload due to the duplication of material in each register and the frequent changes in data entry protocols ^36^. Adapting health care registers to capture the needed information on various reporting forms is also found in other African countries and points to the need to improve and make the health reporting system more flexible to capture multiple service provision in one visit.

In addition, while the innovation shown by the individual health workers in adapting the standard family planning form is important to recognize, it is not a long-term solution to improve the quality, ease, or time requirement of reporting. As in many low-resource countries, some areas of health care provision are supported by donor agencies that fund specific programs which often require additional reporting and documentation. This data should ideally be taken from existing data collection systems but often requires the creation of additional, parallel documents ^37, 38^.

With all the challenges noted above, there is a need to streamline indicators related to maternal health and contraception. One example of this is the FP2020 initiative and the Track20 project, which monitors progress of achieving the FP2020 goals. These goals include increasing modern method users by an additional 120 million women between 2012 and 2020 in the world’s 69 poorest countries, which includes Togo ^39^. The Track20 project aims to reduce the need for heavy reliance on large national household surveys and instead use estimates of data collected through the public and private sector on specified family planning indicators ^39^. Track20 uses a set of core indicators which were selected through a systematic, consultative process to allow for data-driven decision making by countries and measurement of how well individual needs are met ^39^.

### Future research questions

Numerous areas require further research into assessing and improving the reporting systems of integrated family planning programs. Time allocation studies of health care providers could show the actual burden on health workers for each type of task and can demonstrate specific areas that may be appropriate for streamlining, including technologies and task shifting that could reduce time burdens. A study of task shifting was undertaken in Botswana involving the creation of a new cadre of health worker, the Monitoring and Evaluation District Officer. These individuals were trained on the job for their tasks ^40^. After 3 years on the job, data quality had improved, there was increased use of data for disease surveillance, research, and planning, and nurses and other health professionals had more time to focus on the clinical components of their work ^40^. If such task shifting were to be scaled up, health care worker efficiency could improve and burnout could decrease.

Further research is needed to investigate the most effective ways to improve monitoring and evaluation systems themselves, especially in relation to integrated reproductive health programs. A study in Mozambique of a Health Management Mentorship (HMM) program to strengthen health systems in 10 rural districts analyzed change in 4 capacity domains after one year of the program: accounting, human resources, monitoring and evaluation, and transportation management. All the domains except for monitoring and evaluation showed improvement over the one-year mentorship program. The authors of this study noted that challenges included constantly changing program targets and objectives, continually being in a “crisis mode” (constantly trying to catch up on reporting or needing reports in a short period of time) which did not allow time to set up efficient systems, and unavailability of key program staff due to the frequent out-of-office trainings ^41^.

This finding shows that monitoring and evaluation systems are difficult to improve even with the deployment of additional resources specifically for this purpose due to the inherent constraints. Implementation research to improve the functionality and sustainability of monitoring and evaluation within the health system, perhaps using a collaborative quality improvement approach, should be considered.

### Programming and policy recommendations

Recently, the World Health Organization published results from a five-country intervention to strengthen measurement of reproductive health indicators which aimed to improve national information systems for routine monitoring of reproductive health indicators ^42^. Activities within this intervention included revising, standardizing, and making consistent the existing reproductive health indicators gathered through routine systems and building capacity in data collection methods through training and supervision in pilot sites. The country teams reorganized and updated existing monitoring and evaluation frameworks. Challenges encountered even in this focused effort included frequent changes in staffing, delays from administration such as slow response times to updating systems and competing priorities for staff time for implementing reporting improvements. Thus, even with focused intervention it is challenging to streamline and harmonize monitoring and evaluation systems related to reproductive health.

The main recommendations for policy and programming in the Togolese context include consolidating reproductive data for health indicators and reducing provider workload for reporting, especially reporting integrated reproductive health services. This could include adopting electronic data management systems at the health facility level. Currently the largest task of recording integrated family planning is placed on the health care workers, who have adapted health registers to capture the requested information in monthly reports, but this requires extra work, memory, and creativity on the part of the health care worker. When reporting forms are developed they must be standardized to correspond with the associated health register. The number of times the health provider must enter, and re-enter data needs to be reduced.

### Limitations

One main limitation of the health facility assessment included that health facilities in the purposive sample were all affiliated with *AgirPF* and located in urban areas. Another challenge is that the data are cross-sectional and therefore only provide a snapshot of the current monitoring and evaluation system. Lastly, there were challenges associated with photographing registers; however photographs of available registers were only used to illustrate the kinds of adaptations undertaken by health care workers.

Limitations of the key informant interviews included potentially not understanding all the possible perspectives of the informants, differences in the interviewer’s methods for probing and what areas were focused on in each interview, and potential response bias as the study was conducted under the auspices of *AgirPF*.

## Conclusions

Monitoring and evaluation systems are fraught with implementation challenges that affect the quality of data used in patient care, planning, and policy, especially in relation to recording integrated health services. This is a reality not only in Togo but also in other countries. There is a need for a concerted, collaborative effort on the parts of national governments and global partners to address challenges to improve monitoring of integrated health services.

## Data availability

### Underlying data

Due to restrictions of access to data outlined in the research participant agreement approved by the Togolese national ethics committee (CBRS), the datasets generated and analyzed during the study are not publicly available. Readers can access data by contacting the Lillian Carter Center for Global Health and Social Responsibility at the Nell Hodgson Woodruff School of Nursing at Emory University (lcc@emory.edu). Data will only be shared with researchers for reanalysis and grant proposals.

### Extended data

Figshare: Study Documents Agir03. https://doi.org/10.6084/m9.figshare.7823651.v1 ^21^.

This project contains the following extended data:

- Study documents including the protocols, consent forms, health facility assessment forms, and semi-structured interview guides

Data are available under the terms of the Creative Commons Attribution 4.0 International license (CC-BY 4.0).
