# Supplementary material for: ‘ It is a question of determination’: a case study of monitoring and evaluation of integrated family planning services in urban areas of Togo
Source: Gates Open Res. 2019 May 1;3:1451. [Version 1] doi: 10.12688/gatesopenres.12944.1 (PMC6784308; doi:10.12688/gatesopenres.12944.1)
Supplement: Peer review history

# Peer review: Reviewer response for version 1

**DOI:** 10.21956/gatesopenres.14048.r27940

**Published:** 2019-10-07

**Authors:** Karen Hardee

**Affiliations:** 1 What Work Association, Arlington, VA, USA

This is an important and well written paper about the complexities of service delivery reporting systems for monitoring integrated family planning services.  The paper uses data from the AgirPF project in three urban areas of Togo to trace the expected and actual system for recording family planning integrated with postabortion and postpartum services in 25 health facilities and through 41 key informant interviews. While the focus is integrated services, the findings are applicable to reporting systems for any services and provide a cautionary note for programming that does not pay adequate attention to or funding for M&E. For example, the finding that the relevant registers for PAC are not located in the same place is instructive for understanding the complexities of reporting. The finding that providers are overwhelmed with so much reporting for different purposes is unfortunately not new. The use of visuals to show the workarounds that service providers do in order to record needed indicators is useful for those working on improving reporting of service statistics. I was struck by Figure 4 showing that most monthly reports are sent to NGOs – are those implementing partners funded by donors?  Are they contributing to the complexity and work for providers by, as the authors say, “If an official register did not exist for a given services, sometimes NGO workers involved in integration projects would tell health workers to create an unofficial register for the service using a notebook and pen, inserting columns for recording information.” 

 I did not find the paragraph about FP2020 and Track20 in its current version so relevant to this paper. Service statistics constitute a small component of the data that go into FP2020’s 18 core indicators – and few of the 69 countries have strong enough systems to produce data of sufficient quality to use in the reporting. With that said, I think Track20 is doing interesting work with countries on their routine health systems – particularly to rationalize indicators. Rather than include a summary paragraph about Track20, I suggest that the authors contact Track20 to find out what they are doing related to routine health information systems – so the paragraph can be better tailored to the paper. 

 I was surprised to see no mention of the work of MEASURE Evaluation, which has for decades been working on improving M&E systems, including routine health information. As one example, through JSI, MEASURE Evaluation has worked on PRISM (Performance of Routine Information System Management), that would be useful to review. Another resource is RHINO, the Routine Health Information Network ( https://www.rhinonet.org/). And PEPFAR, with its focus on reporting indicators, has generated lessons learned for strengthening routine health information systems. I suggest that the authors check these resources to strengthen the section of the paper on how to make improvements in the reporting system. 

 Also, it would be good for the authors to say something about the official process of revising the components of routine health information. For example, who has the authority to make changes to the system? How often are registers reviewed and revised? By who? The authors indicate that some respondents recommended computerized reporting with tablets. Who would have the authority to make this change to the M&E system? Any implementation research carried out to strengthen reporting of integrated family planning services will need to acknowledge the official process for making changes – otherwise, it might just be creating more workarounds.

I confirm that I have read this submission and believe that I have an appropriate level of expertise to confirm that it is of an acceptable scientific standard.

# Peer review: Reviewer response for version 1

**DOI:** 10.21956/gatesopenres.14048.r27109

**Published:** 2019-05-28

**Authors:** Jane T. Bertrand

**Affiliations:** 1 Tulane University School of Public Health and Tropical Medicine, New Orleans, LA, USA

This article addresses the challenge of monitoring and evaluating integrated family planning services in urban areas of Togo. Specifically, it focuses on the integration of services for family planning, post abortion care, and postpartum care. The findings are based on an assessment of 25 purposively selected health facilities, a review of health services records, and 41 in-depth interviews with different categories of health personnel.

 The article is well written and clearly presented. The photos of the registers are useful in reminding readers of the very basic data collection tools available in these facilities. The photos also reflect the attempts by health providers to do the right thing, in the absence of having the appropriate tools to do so.

 The paper merits publication because of its systematic examination of the issue of monitoring integrated family planning services. With a new interest in postpartum (and in some countries post-abortion) family planning, this methodological issue is very relevant to those working in family planning. It captures a reality found in many health systems in low-income countries, which often is “understood” but not documented.

 The paper effectively communicates the tension between good service provision and timely data collection by low- to middle-level health workers, often poorly paid, who feel overwhelmed by the amount of documentation they are required and expected to produce.

 Missing from the paper is a clear statement of what the authors would consider the short list of most valuable indicators to measure integrated family planning. The service providers developed ingenious “work-arounds” to provide information relevant to this question, as they improvised the use of the available forms. If the researchers had full reign over the data collection, what would they recommend? This is particularly relevant, given their citation of the FP 2020 indicators as an example of a standardized set of metrics that countries could then strive to obtain and report.  Table 2 provides a list of 13 indicators that includes integrated family planning services. However, the list is long, and the authors don’t attempt to identify or prioritize those of greatest relevance to the question. For readers looking to better understand best practices in the area of measuring integrated family planning services, it’s important to learn what this handful of indicators most important in measuring integrated family planning services would be.

 In a related vein, there is lack of a clear path toward a better system. The authors do provide recommendations for improving the quality of service statistics by capturing the data at all levels and reducing the reporting burden on service providers. Yet the very circumstances that created the problems in the current data collection system are fairly ingrained in weak health systems (in Togo and elsewhere). Can the authors provide concrete examples of where their recommendations have actually paid off in other countries?

 The following edits would improve the flow and content of the paper:

1. Paragraph 3 under the introduction: replace “in opposition to…” with “in contrast to…”
2. Under procedures/health facility assessment: The authors clearly explain the purpose of the sample of 25 health facilities. It would be useful to have an approximate estimate of the universe of facilities from which the 25 were selected.
3. Under results/health facility assessment: it is not intuitively clear why the researchers took half as many facilities from Sodoké as from Kara, when in the previous section they explain that the population of Sodoké is slightly larger than that of Kara.
4. Figure 4. The idea behind this graph and the explanation underneath it do not clearly communicate what it is intended to show. For instance, what the expected/preferred route of transmission?
5. On page 10, under “uses of family planning data reported,” the authors make the very salient point that “only six of 41 informants… noted that they use collected data from the health facilities themselves to inform their work and programming.” The author should further develop this issue in the discussion.

With these minor edits, I recommend indexing.

I confirm that I have read this submission and believe that I have an appropriate level of expertise to confirm that it is of an acceptable scientific standard.
